# Identification of non‐glandular trichome hairs in cannabis using vision‐based deep learning methods

**DOI:** 10.1111/1556-4029.70058

**Published:** 2025-04-18

**Authors:** Alon Zvirin, Amitzur Shapira, Emma Attal, Tamar Gozlan, Arthur Soussan, Dafna De La Vega, Yehudit Harush, Ron Kimmel

**Affiliations:** ^1^ Computer Science Department Technion – Israel Institute of Technology Haifa Israel; ^2^ The Division of Forensic Sciences National Police Headquarters Jerusalem Israel; ^3^ Faculty of Electrical and Computer Engineering, Technion – Israel Institute of Technology Haifa Israel

**Keywords:** cannabis detection, colorimetric chemical tests, computer vision, cystoliths, deep learning, non‐glandular trichomes, synthetic cannabinoids

## Abstract

The detection of cannabis and cannabis‐related products is a critical task for forensic laboratories and law enforcement agencies, given their harmful effects. Forensic laboratories analyze large quantities of plant material annually to identify genuine cannabis and its illicit substitutes. Ensuring accurate identification is essential for supporting judicial proceedings and combating drug‐related crimes. The naked eye alone cannot distinguish between genuine cannabis and non‐cannabis plant material that has been sprayed with synthetic cannabinoids, especially after distribution into the market. Reliable forensic identification typically requires two colorimetric tests (Duquenois‐Levine and Fast Blue BB), as well as a drug laboratory expert test for affirmation or negation of cannabis hair (non‐glandular trichomes), making the process time‐consuming and resource‐intensive. Here, we propose a novel deep learning‐based computer vision method for identifying non‐glandular trichome hairs in cannabis. A dataset of several thousand annotated microscope images was collected, including genuine cannabis and non‐cannabis plant material apparently sprayed with synthetic cannabinoids. Ground‐truth labels were established using three forensic tests, two chemical assays, and expert microscopic analysis, ensuring reliable classification. The proposed method demonstrated an accuracy exceeding 97% in distinguishing cannabis from non‐cannabis plant material. These results suggest that deep learning can reliably identify non‐glandular trichome hairs in cannabis based on microscopic trichome features, potentially reducing reliance on costly and time‐consuming expert microscopic analysis. This framework provides forensic departments and law enforcement agencies with an efficient and accurate tool for identifying non‐glandular trichome hairs in cannabis, supporting efforts to combat illicit drug trafficking.

**Highlights**

- Automated AI method to distinguish genuine cannabis from non‐cannabis trichomes.
- Integration of advanced deep learning approaches for object recognition and classification.
- Application of a three‐stage decision mechanism for high‐performance identification.
- Verification of the system on a uniquely curated microscopic image dataset.
- Potential to reduce reliance on costly and time‐consuming tests by a laboratory specialist.

## 1 INTRODUCTION

The term “cannabis” originates from the Scythian and Assyrian languages, with the root K‐N‐B (pronounced ka‐na‐ba or qu‐nu‐bu) entering both Indo‐European and Semitic languages, making it one of the oldest surviving root words [1, 2]. Fabrics from cannabis hemp were woven thousands of years ago in several distinct civilizations, going back into pre‐historical eras [3, 4]. The first record of medical and hallucinative usage of cannabis is found in the *Pen‐Ts'ao Ching*, a Chinese Pharmacopeia compiled in the first century AD, but most likely passed as oral traditions centuries earlier [5, 6].

Cannabis is an herbaceous dioecious plant belonging to the order Urticales and the Cannabaceae family. The taxonomic description of the cannabis genus is somewhat controversial; “*sativa*” is considered de facto the sole species of the genus. Although other species such as “*indica*” and “*ruderalis*” have been proposed, these are now widely regarded as varieties rather than distinct species [7].

Cannabis contains a peculiar class of phytochemicals known as phytocannabinoids. From a chemical point of view, cannabinoids are meroterpenoid compounds with a basic structure of a resorcinyl moiety with different isoprenyl, alkyl, or aralkyl side chains. Tetrahydrocannabinol (THC) and the corresponding acid tetrahydrocannabinolic acid A (THCA‐A), cannabidiol (CBD), cannabinol (CBN), and cannabidiolic acid (CBDA) are the main components of this class; altogether, over 200 different cannabinoids have been isolated from the leaves of *Cannabis sativa* [7].

During the early 2000s, synthetic cannabinoids began to appear in the drugs market. Synthetic cannabinoids are functionally similar to Δ9‐tetrahydrocannabinol (Δ9‐THC), the main psychoactive substance in cannabis. They bind to the same cannabinoid receptors in the brain and other organs as THC. Most blends consist of synthetic cannabinoids sprayed onto inert plant matter like damiana leaves, which could then be smoked as cigarettes (“joints”) [8, 9]. In the plant base of these mixtures, there are hairs that differ in appearance from the non‐glandular trichome hairs in cannabis.

Approximately, 70% of all cases examined in the National Drug Laboratory of the Israeli police are cannabis or cannabis products. The annual amount examined surpasses several tons. To identify cannabis and cannabis products, the National Drug Laboratory uses the internationally recommended identification methods, including inspection of microscopic views of non‐glandular trichomes [10, 11, 12] and two chemical color tests, the Duquenois‐Levine test [12, 13, 14, 15, 16] and the Fast Blue BB test [17, 18]. Identification of cannabis plants and cannabis substances is carried out in a drug laboratory hosted by the police, and is jointly determined by these colorimetric tests and an expert human observation of microscopic images.

The Duquenois–Levine test is part of the analytical scheme for marijuana analysis [13, 14, 15, 16, 17]. A three‐part solution, the Duquenois–Levine test reacts with the cannabinoids in marijuana to produce a purple bi‐layer as a positive result. The Duquenois reagent is composed of ethanol, acetaldehyde, and vanillin. In an acidic environment, normally achieved through the use of concentrated hydrochloric acid, the reagent will react with the free position para to the phenol group of the cannabinoid. However, many compounds contain a phenol group with a free para position, creating the potential for false positives. The Levine modification eliminates this potential through the addition of chloroform. Only molecules with a long aliphatic chain are able to cross into the chloroform layer. THC has a five‐carbon chain on the number 3 carbon, allowing for this transition. The Fast Blue BB test [17, 18] is a colorimetric test in which the development of a reddish color in a basic medium indicates the presence of cannabinoids such as Δ9‐THC, cannabinol (CBN) and cannabidiol (CBD). Fast Blue BB salt produces stable diazo compounds with cannabinoids in alkaline media.

**FIGURE 1:**
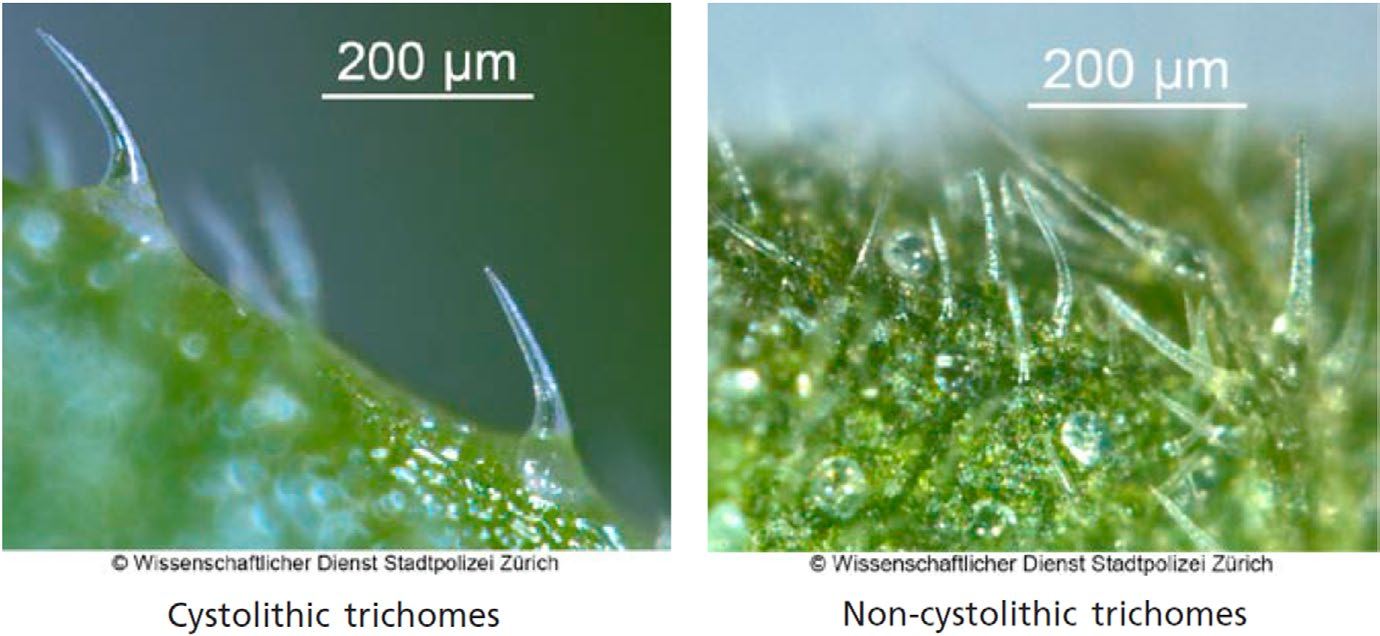
Examples of non‐glandular trichomes, cystolith (left), and non‐cystolith (right). Microscopic view with magnification 40. From Wissenschaftlicher Dienst, Stadtpolizei Zürich, Switzerland, with permission.

**FIGURE 2:**
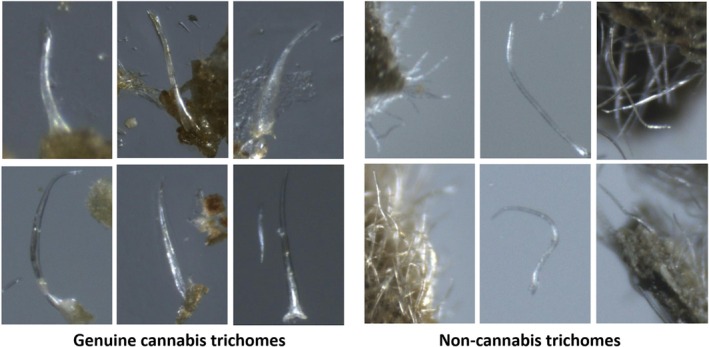
Samples of non‐glandular trichome hairs in cannabis (left) and non‐cannabis trichomes (right) from the dataset collected during this research. The images displayed here are cropped from the original, larger images. Typically, the genuine trichomes have a bear‐claw shape, whereas the non‐cannabis trichomes are narrower and more densely distributed.

Manual identification of cannabis is performed by examining physical characteristics of the plant via microscopic images. An important characteristic of cannabis is its trichomes (i.e., hair‐like projections from a plant epidermal cell) which are minuscule structures on the surface of the plant [19]. Both types of trichomes, non‐glandular trichomes and glandular trichomes, appear on the leaves and can be observed with a magnification factor of 40. Non‐glandular trichomes are numerous, unicellular, rigid, curved hairs, with a slender, pointed apex. Characteristic bear‐claw‐shaped trichomes are found only on the upper (adaxial) surface of cannabis leaves. These trichomes may sometimes contain calcium carbonate crystals (cystoliths) visible at their base (cystolithic trichomes) as shown in Figure 1. When trying to differentiate between cannabis and non‐cannabis plant material, the trichomes can be a good criterion since non‐cannabis plant material lacks the peculiar bear‐claw‐shaped trichomes. Non‐cannabis trichomes are less curved than those on genuine cannabis, and usually have more of a needle shape, as shown in Figure 2.

Physical examination uses the morphological characteristics (macroscopic and microscopic) of the plant in order to determine its type. Macroscopic characteristics include its color, height, leaf structure, stem build, and inflorescence shape. Microscopic characteristics are the trichomes that can be observed with microscope magnification. Both glandular and non‐glandular trichomes appear on the cannabis plant, each located in a different area and having entirely different structures [12]. Although morphological feature identification of the cannabis plant is useful for its classification, physical observations are not reliable enough, and chemical examinations are required for accurate cannabis identification. These are generally based on chromatography, a biophysical technique that enables the separation, identification, and purification of the components of a mixture [20]. Some of the popular ones are gas chromatography–mass spectrometry (GC–MS) and high‐performance liquid chromatography (HPLC) [21]. Color tests are quicker and cheaper chemical methods, performed by placing chemicals into a test tube with a sample of the suspected drug and drawing conclusions from the color changes that indicate the presence of a THC compound [22]. The most common cannabis color tests are the Duquenois–Levine and Fast Blue BB, described previously.

Developing an automated method for identifying non‐glandular trichome hairs under a microscope will allow the police to cut costs and reduce the time required to complete the expert opinion report. Consequently, they could be much more efficient with their limited law enforcement resources. Increased efficiency means improved ability to support investigation requirements, and therefore, eventually indict drug offenders. Our hypothesis here is that a deep learning‐based system can indeed be implemented for fast and accurate detection of cannabis versus non‐cannabis trichomes, on par with chemical tests and expert human observation.

We applied several deep learning approaches for distinction between “*the real and the fake”* (i.e., between genuine cannabis and non‐cannabis plant material). Two approaches were tested and compared: image classification and object recognition. We also designed a special decision strategy, integrating the classifiers and the object detectors, resulting in a high identification accuracy of 97.61%. Our main contributions are: (1) Development of an automatic deep learning vision‐based system for cannabis identification; and (2) Verification of the system on a uniquely collected image dataset. To the best of our knowledge, this is the first such a system. We expect that following further data accumulation and testing, it could be used in forensic analysis in the near future. The complete software package and the collected dataset, images, and annotations, are openly and freely available at the authors' github repository (https://github.com/alongitzv/cystolith‐detection).

## 2 RELATED WORK

The use of deep learning and computer vision in various domains is growing rapidly. In many cases, it has been demonstrated that it can replace manual processes by faster, more accurate automatic processes, sometimes surpassing expert human observation. Image‐based deep learning has been used to classify diseases and nutrient deficiencies in cannabis [23, 24], but, until now, has not been exploited for identification of cannabis by appearance of non‐glandular trichome hairs. Current methods for cannabinoid analysis are based on a combination of physical and chemical examination, as recommended by the Scientific Working Group for the Analysis of Seized Drugs (SWGDRUG) [17].

Classification of plants and plant parts is an important task in the worlds of plant cultivation and agricultural research. Improving it by using computer vision tools can benefit many industries. Several works utilized deep learning techniques for plant identification and classification, mainly focusing on analyzing the leaves, since they tend to convey information relevant to the plant's growth stage and possible disease or stress condition [25, 26, 27, 28, 29]. Studies that used *Convolutional Neural Networks* (CNN) [30, 31] and *You Only Look Once* (YOLO) [32, 33] were able to achieve high accuracy for plant and plant disease classification, demonstrating the ability of these methods to extract useful agricultural information from the images.

In the field of criminology, several studies have been conducted to explore how computer vision and deep learning can make crime investigations more efficient. Various methods and tools have been used for crime prediction [34], crime scene analysis [35] and even forensic identification from police sketches [36]. Yet, insofar as we are aware, no previous work utilized microscopic images for the task of cannabis identification, specifically for classification of non‐glandular trichome hairs in cannabis vs. non‐cannabis plants sprayed with synthetic cannabinoids.

## 3 MATERIALS AND METHODS

For this research, a special image dataset was collected and curated, consisting of several thousand microscopic images of cannabis hairs and hairs of plant material sprayed with synthetic cannabinoids. Most of the plant material were inspected in this research, cannabis and non‐cannabis were collected “in the street” by the police as part of law enforcement activity. In many cases, the material was already packaged and ready for distribution in the market. All photographed plant hairs underwent the two specified tests (Duquenois‐Levine and Fast Blue BB), as well as the drug laboratory expert test for the affirmation or negation of cannabis hair (non‐glandular trichomes), results of which were used as ground‐truth labels for the machine learning algorithms. Most of the images were annotated by rectangular bounding boxes surrounding some of the glandular hairs, serving as training data for the object detection modules. The object detection models were trained to detect two types of objects: non‐glandular trichomes (hair from the cannabis plant) and hair from non‐cannabis plants assumed to be sprayed with synthetic cannabinoids. The underlying assumption is that deep neural networks can indeed identify the morphological structure of the trichomes and differentiate the “real” (cannabis hair) from the “fake” (non‐cannabis hair).

We applied several deep learning approaches to identify the hair of cannabis versus the hair of a non‐cannabis plant. Two different binary classifiers were implemented, a basic *Convolutional Neural Network* (CNN) [37] and an advanced model known as *Deep Layer Aggregation* (DLA) [38]. These classifiers operate on the image as a whole, outputting a single label (real or fake) for the entire image. Next, we applied modern object detection methods aimed at identification of the trichome hairs appearing in the images. To this end, we utilized and compared two competing state‐of‐the‐art methods, *You Only Look Once* (YOLO) [39] and *DEtection TRansformer* (DETR) [40]. Finally, we designed a special decision strategy, integrating the classifiers and the object detectors.

### 3.1 Data collection

The plants examined were those obtained from various cases under investigation and indictment. The plants were collected from different apartments or greenhouses where they were suspected of being cannabis plants. In some cases, the plant material was already packaged as drug parcels intended for distribution. For plants that were determined not to be cannabis through laboratory testing using various solvents and microscopic analysis, which showed no evidence of cannabis trichomes, there was suspicion that these plants had been sprayed with synthetic cannabinoids. Synthetic cannabinoids typically consist of various chemical compounds designed to mimic the effects of THC, the active compound in cannabis. Criminals attempt to spray different substances that vary from case to case and plant to plant.

In this study, we did not analyze the chemical composition of the plants in the numerous cases where laboratory results were negative for cannabis (i.e., they did not contain THC) or showed hairs/trichomes that did not resemble those of cannabis. In each case, the plants were examined by an expert using the two mentioned chemical tests and a microscopic visual analysis to provide expert opinions for investigative teams and courts regarding whether the plant material was cannabis (defined as a dangerous drug under the Dangerous Drugs Ordinance) or not.

The plant material's texture is typically in the form of broken leaves or flowers, some of which are evenly ground up. A small amount, ~26 mg (a minimal quantity is sufficient for qualitative rather than quantitative tests), is taken and transferred to a Petri dish. The material is then examined under a microscope with a zoom magnification level between 20× and 128× to identify the characteristic cannabis trichomes. This process involves a visual identification of cannabis trichomes, which are unique to the cannabis plant, using a microscope for enhanced resolution. This method is commonly used for qualitative testing, ensuring that the sample contains cannabis material based on the presence of its distinctive features, such as glandular trichomes.

### 3.2 Data processing

**FIGURE 3:**
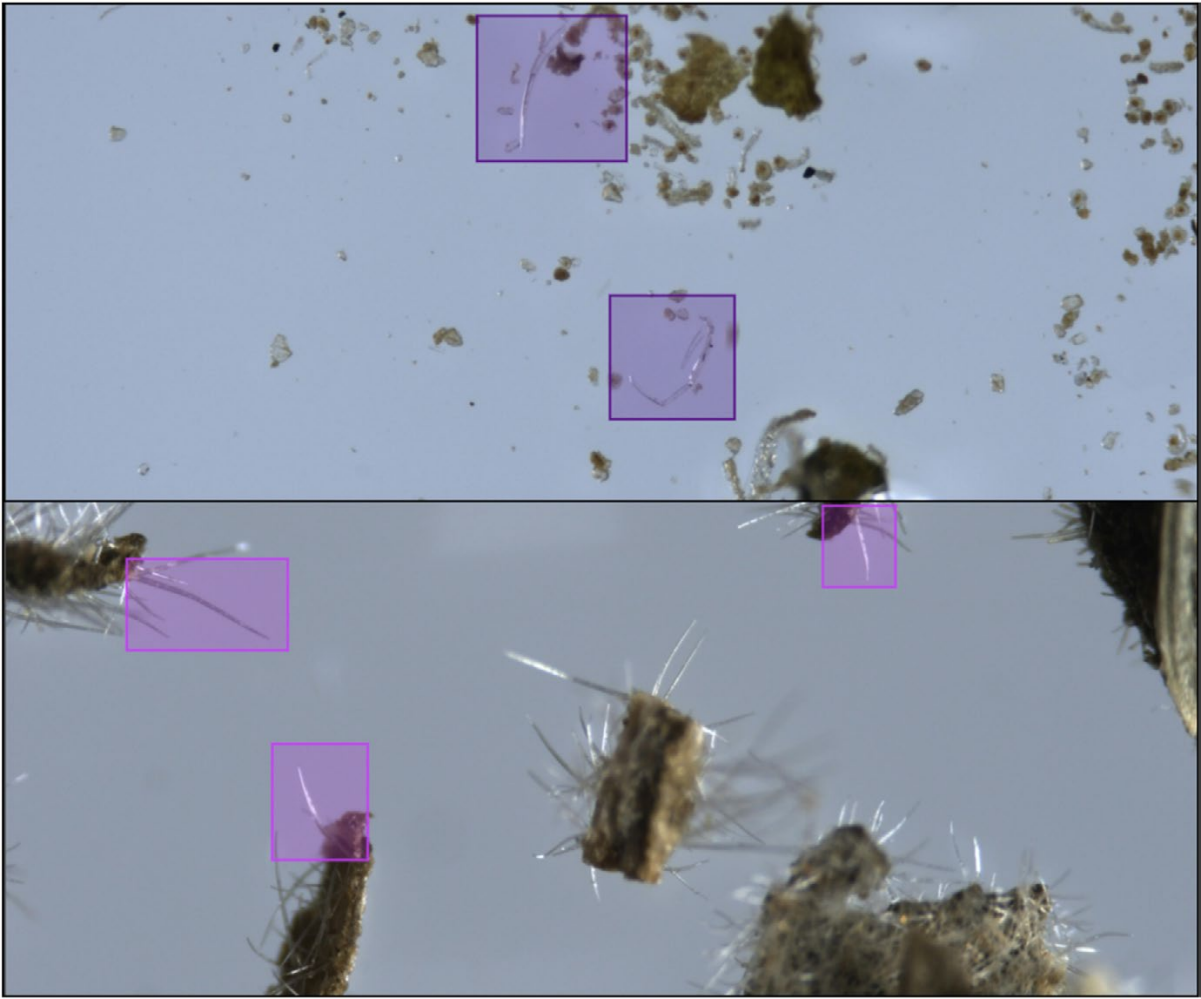
Samples of original images and bounding box annotations from the data collected, used for training the object detectors. Top – genuine, non‐glandular trichome hairs in cannabis; bottom – trichome hairs on non‐cannabis plants.

The dataset consists of 2826 microscopic images, 1286 of genuine cannabis and 1540 of non‐cannabis material. These were collected from more than 2000 different samples. In most cases, each sample was photographed once, but in some cases twice to achieve better focus on different parts of the material. The exact number of sampled plants is unknown, since the material was collected by the police as part of law enforcement activity against illicit drug trafficking. As such, most of the plant material was collected in packages ready for distribution, already ground, crushed, and minced, and in some cases, pulverized and powdered. We used an OLYMPUS SZX16 microscope [41] and a Pixelink PL‐D799CU camera with Pixelink software [42], with image resolution of 4096 × 1710 pixels and standard jpg RGB color format. To annotate the trichome hairs, we used MakeSense, a free open‐source visual software tool [43]. Most of the images were annotated with rectangular regions of interest (ROI) surrounding the trichomes, whether “real” or “fake”, usually 1–2 bounding boxes per image. Bounding boxes are marked by two mouse clicks determining the object's upper‐left and lower‐right corners. The annotation software allows zooming in and dragging mode, thus enabling a tight enclosure of the objects. Each annotation contains five parameters; the first specifying the object's semantic label (“real” or “fake”), and four numerical values indicating the *x*,*y* location of the region's center, and width, height of the rectangular bounding box, normalized with respect to the image size. These ROIs vary in size, between 45 × 35 pixels and 1520 × 800 pixels; most are smaller than 512 × 512, the average size being 287 × 279 pixels for cannabis trichomes and 339 × 337 pixels for non‐cannabis trichomes. The total number of annotated bounding boxes was 3158, 1461 of cannabis trichomes and 1697 of non‐cannabis trichomes. Samples of original images and bounding box annotations are presented in Figure 3. It should be noted that this dataset contains a variety in terms of lens focus, illumination and reflectance, artifacts, and amount of relevant foreground material versus irrelevant or noisy background in the images. At this stage of data acquisition and trichome annotation, the raw images themselves were used, without any preprocessing.

### 3.3 CNN classifier models

CNN classifiers are deep learning algorithms famous for their ability to extract representative information from image data with minimal preprocessing. CNNs have demonstrated outstanding performance in image recognition and classification tasks, mainly due to their structure: A sequence of computational layers enables stratified representations of the data, from low‐level characteristics to higher‐order abstract features. A key aspect of network optimization is that parameter values are iteratively fine‐tuned, or “learned,” as opposed to being engineered or “hand‐crafted,” as in traditional image processing algorithms [44].

**FIGURE 4:**
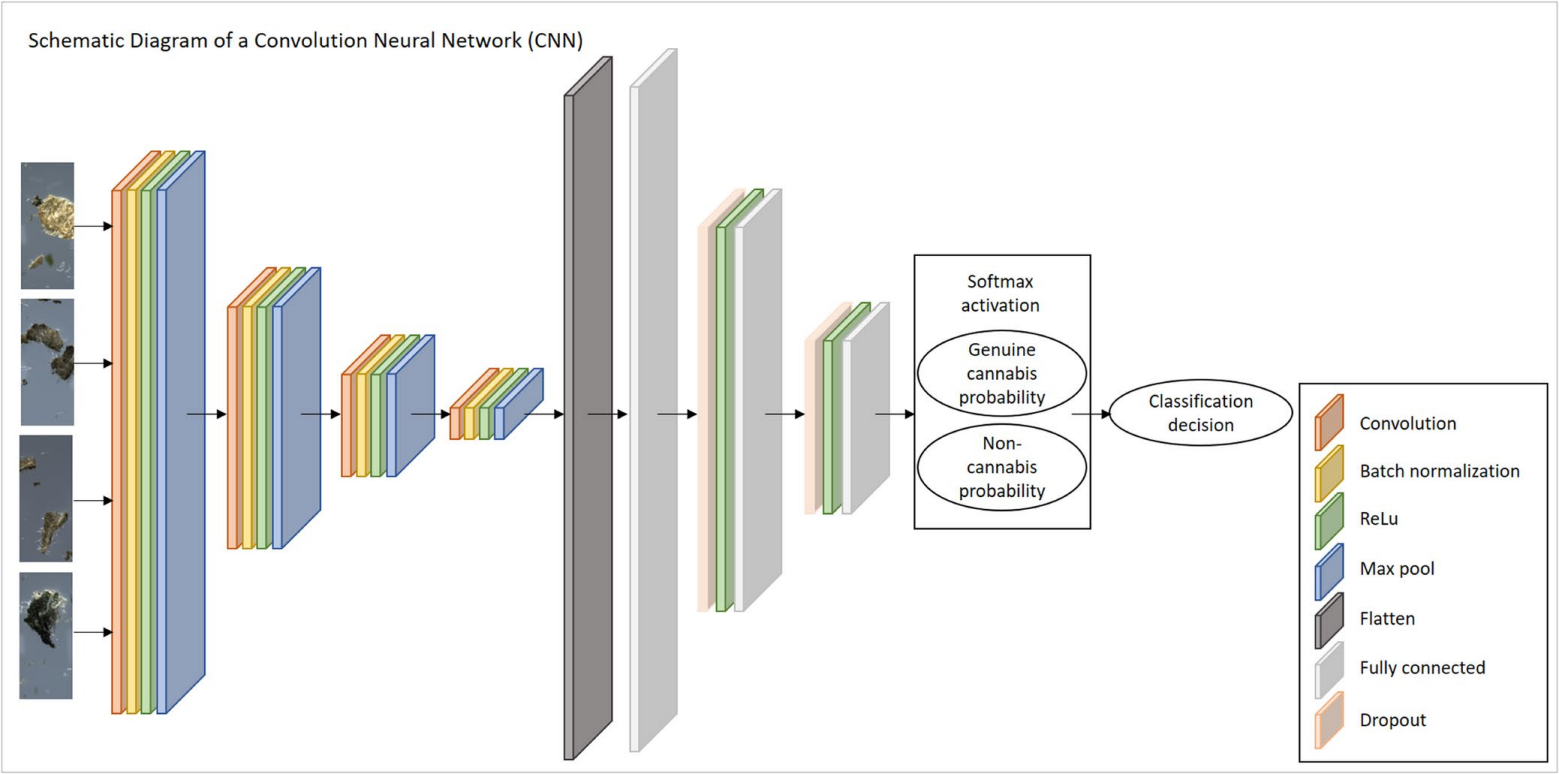
Architecture of the basic CNN model. The network is designed for binary classification and accepts as input color images of size 1024 × 428. It consists of four convolutional blocks, each comprising a convolutional layer, batch normalization, a ReLU activation, and max‐pooling for down‐sampling. The feature map sizes are progressively reduced across layers, starting with 16 channels and ending with 128 channels in the final block. The output of the last pooling layer is flattened and passed through a fully connected module with two intermediate layers. Each fully connected layer is followed by a dropout for regularization and a ReLU activation. The final layer is a fully connected output layer with two neurons corresponding to the binary classification task.

We used two different CNN models for binary classification of the images. The first is a basic CNN implementation, widely used in image classification. It consists of four blocks of layers, each block composed of a convolution layer, a batch normalization layer, a rectified linear unit (ReLU), and a max‐pooling layer. These are followed by three fully connected linear layers with dropout and ReLU activation between them, and finally a softmax operation that outputs a probability score for each class. The class with the higher probability is selected as the network's prediction of the image label. Figure 4 depicts a schematic view of this particular CNN structure. Images inputted to this network are rescaled by a factor of 0.25 on each axis, into 1024 × 428 pixels, using bi‐cubic interpolation. The first four (convolutional) blocks output 16, 32, 64, and 128 feature maps, respectively. All convolution kernels are 3 × 3, default batch normalization and ReLU are used, and all max‐pooling layers use a 2 × 2 pixel kernel. The input and output number of nodes in the three fully connected layers are (128 × 26 × 64, 1024), (1024, 32), (2, 32), respectively. The two dropout layers apply a 0.25 probability.

**FIGURE 5:**
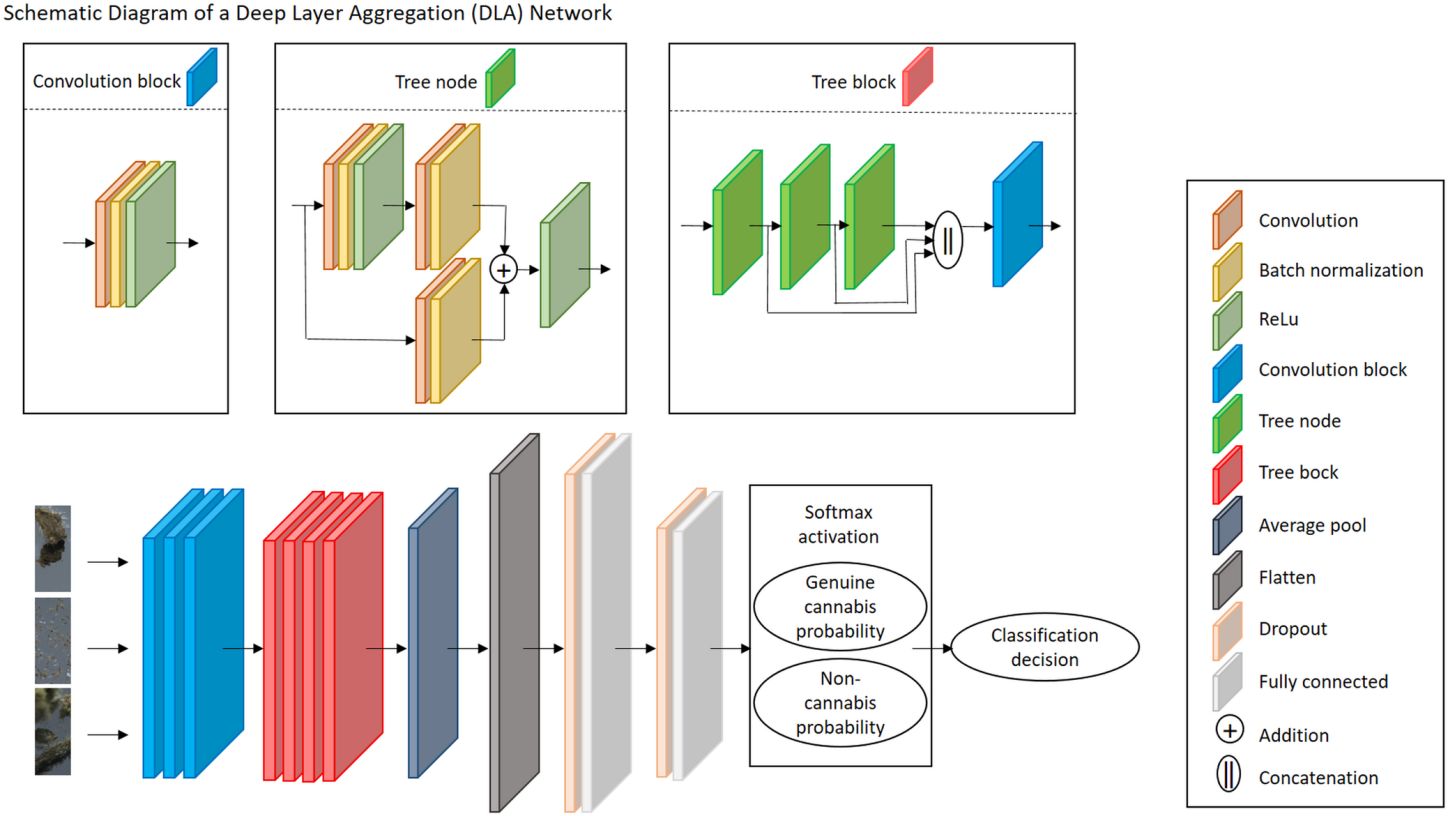
Architecture of the deep layer aggregation (DLA) network. This network introduces a hierarchical structure with tree modules to aggregate features across multiple scales. Input color images with 512 × 214 pixel resolution are processed through three initial convolutional blocks. Each block consists of a convolution, batch normalization, and ReLU activation, outputting 16, 32, and 32 feature maps, respectively. The hierarchical core of the DLA consists of four tree modules. Each module contains multiple tree nodes, which aggregate features from previous layers using addition and/or concatenation operations via skip connections. The feature map dimensions progressively increase, with the tree modules producing 64, 128, 256, and 512 feature maps, respectively. After feature aggregation, the network applies a global average pooling layer, reducing the spatial dimensions of the feature maps. Two fully connected layers are used for classification. Dropout layers are incorporated after each fully connected layer to reduce overfitting. A final softmax activation outputs the probability scores for each class, with the class having the higher probability selected as the predicted label.

The DLA implementation consists of similar layered blocks (convolution, batch normalization, and ReLU), but with a tree structure, as opposed to a completely sequential arrangement. This structure utilizes skip connections, residual operations, and hierarchical representations by adding and concatenating outputs from previous layers as inputs to next layers. The DLA network is more densely populated with blocked layers compared with the basic CNN, followed by a composition of fully connected, dropout, and softmax activation, ending with a probability score for each class. A schematic diagram of the DLA's architecture is presented in Figure 5. Images inputted to this network are rescaled by a factor of 0.125 on each axis, into 512 × 214 pixels, using bi‐cubic interpolation. The first three blocks consist of convolution, batch normalization and ReLU layers, without any pooling. These block output 16, 32, and 32 feature maps, respectively. All convolution kernels are 3 × 3; default batch normalization and ReLU are used. These are followed by four tree blocks, composed of hierarchical structure of multiple nodes consisting of convolution, normalization and ReLU layers. These nodes are connected via skip connections with addition and/or concatenation operations linking them. The four tree nodes output 64, 128, 256, and 512 feature maps, respectively. These are followed by an average pooling layer with a 4 × 4 kernel, and two blocks of dropout and fully connected layers, dropouts with 0.25 probability and fully connected layers with (512 × 3 × 32, 32), (2, 32) input and output nodes, respectively. Finally a softmax operation outputs a probability score for each class, and the class with higher probability is selected as the DLA's prediction of the image label.

### 3.4 YOLO and DETR object detection models

YOLO is a single‐shot model for detection and localization of objects in images that uses a robust CNN architecture [45]. While previous object detection models used a pipeline that went over the input image multiple times, YOLO was the first to provide real‐time detection using a single pass on the input images [39]. The model divides the image into a fixed grid and calculates for each grid cell the probability score for objects' location and class label. Though YOLO predicts multiple bounding boxes per grid cell, it selects one object prediction per bounding box during training. The YOLO version chosen for this study is YOLO‐V4, which provides faster and more accurate detections compared with other versions [46]. We used Bochkovskiy's yolov4‐darknet implementation [47].

DEtection TRansformer (DETR) [40] is a type of vision transformer (ViT) [48] that adapts a self‐attention mechanism [49] that replaces convolutional operations in image classification and recognition tasks. The basic attention mechanism consists of a sequence of linear embeddings of image patches and non‐linear embeddings of the patches' positional encoding. DETR uses multiple attention blocks in an encoder‐decoder structure. With a flexible architecture that is relatively simple to implement, DETR reaches high accuracy and impressive run‐time performance on standard benchmarks. We used the roboflow‐huggingface implementation [50].

**FIGURE 6:**
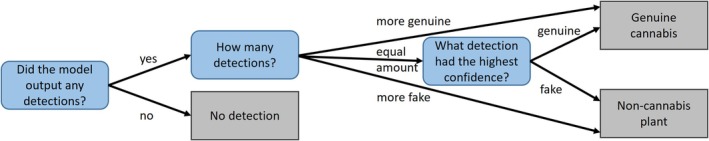
Decision scheme for class label prediction from the object detector models. The amount of labeled objects that were detected determines the class prediction. If equal, the detected object with the highest confidence determines the image's label. In cases with no detections, the class remains undecided, and the prediction is considered an incorrect result.

Object detectors do not classify images; they output bounding boxes surrounding candidate sought‐for objects, with a label and probability score attached to each such object. Therefore, a decision scheme for classifying the image is still required. Basically, if more objects of a certain type are detected, prediction of the image label is straightforward. In this binary classification task, if more objects are detected from one class than the other, the image is labeled as belonging to that class. If the number of detected objects is equal between the two classes, the label is determined by the object with the highest probability score. If no trichomes are detected, the output is labeled as “no detection,” which is treated as a false prediction. This decision scheme is depicted in Figure 6. The accuracy of these models was assessed according to the percentage of true detections out of all detections.

### 3.5 The composite method

**FIGURE 7:**
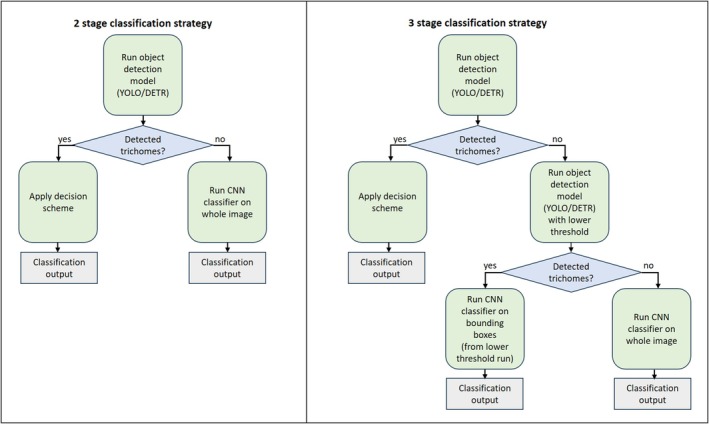
Two suggested options for multi‐stage decision strategies integrating object detectors with image classifiers. In both cases, the first stage is to run an object detector attempting to detect trichome hairs and label them as cannabis or non‐cannabis trichomes. In the two‐stage strategy (left), if the object detector fails, a whole‐image classifier is then applied. In the three‐stage strategy (right), if the first object detector fails, a lower‐threshold object detector is then run at the second stage, with the objective of outputting more candidate trichomes (having lower confidence levels). Next, if any trichome hairs are detected, a classifier is applied on their bounding boxes; otherwise, a whole‐image classifier is applied.

Since the main objective is to distinguish between two categories, “real” and “fake” cannabis, and not necessarily to obtain details or features appearing in the images, a binary classifier is the first and most obvious choice. However, microscopic images contain much background and artifacts. These affect the models' algorithmic mechanism, and heavily bias the performance and outputs, especially in small datasets (thousands as opposed to millions) as used for this study. This leads us to identify specific object features that are assumed to differentiate cannabis hairs (non‐glandular trichomes) from non‐cannabis plant hairs, namely, the shape of the hairs. As these are not trivial to detect, and in some cases are not detected at all, we propose to combine the two approaches, taking advantage of the strengths of each approach. To this end, we propose two versions of a special purpose decision strategy, integrating a classification model and an object recognition model, implemented via a multi‐stage algorithm. The basic idea is to apply an object detector, and in cases where it fails in confident object detections to apply an image classifier. A more sophisticated option is to use, in case of no detections, a similar object detector trained to a lower confidence threshold, thus outputting more candidate bounding box predictions. The second stage classifier, in this case, operates on the bounding boxes produced by the first stage detector, not on the entire image, thus having a better chance of correct identification due to its focus on image patches which are more informative. Below, we formulate these strategies as algorithmic pipelines. Flowcharts depicting these mechanisms are presented in Figure 7.

#### 3.5.1 Two‐stage decision strategy

Stage 1 – Object detection:

- Apply an object detector to identify trichome bounding boxes and classify them as cannabis or non‐cannabis.
- If successful (i.e., confident detections are made):
  Return the class label based on the detected objects.
- If no confident detections are made:
  Proceed to Stage 2.

Stage 2 – Whole‐image classification:

- Apply an image classifier to the entire image.
- Return the class label predicted by the image classifier.

#### 3.5.2 Three‐stage decision strategy

Stage 1 – High‐confidence object detection:

- Apply an object detector with a high‐confidence threshold to identify trichome bounding boxes and classify them as cannabis or non‐cannabis.
- If successful (i.e., confident detections are made):
  Return the class label based on the detected objects.
- If no confident detections are made:
  Proceed to Stage 2.

Stage 2 – Low‐confidence object detection:

- Apply a similar object detector trained with a lower confidence threshold to generate more candidate bounding boxes.
- If bounding boxes are detected:
  Proceed to Stage 3.
- If no bounding boxes are detected:
  Apply a whole‐image classifier and return the class label predicted by the image classifier.

Stage 3 – Bounding box classification:

- Apply an image classifier to each bounding box generated in Stage 2.
- Aggregate the predictions to determine the final class label.

As part of the three‐stage strategy, a bounding‐box classifier is employed. This classifier is trained on a distinct dataset curated specifically for its role in the inference pipeline. To generate this dataset, we applied lower confidence thresholds to the YOLO/DETR networks, resulting in an abundance of trichome detections. This approach generated over 55,000 detections from the first training split and over 32,000 from the second. These detections included multiple instances of the same trichomes with slightly varying bounding box coordinates, along with numerous non‐trichome patches. However, all detections were derived from images with known ground truth, based on chemical tests, labeling them as either genuine cannabis or non‐cannabis plant material. The bounding boxes formed the foundation for the classifier's dataset, which was created by cropping 512 × 512 image patches centered on the detected trichomes. The DLA network, previously used as a whole‐image classifier, was re‐trained on these cropped patches to function as a dedicated bounding‐box classifier.

## 4 RESULTS

This section presents the results obtained during the study. Following common practice in machine learning tasks, the images were partitioned into training, validation, and test subsets, with an approximate 80%, 10%, and 10% ratio, respectively. Since some of the plants were photographed more than once, the data were separated at the plant level, as opposed to the image level, so that all images of the same plant always appear in the very same subset, eliminating the possibility that the system infers from images of the same data it was trained on. For reliability and cross‐validation purposes, we applied two different training/validation/test data splits. All software experiments were evaluated on the same data splits. Table 1 presents the dataset statistics, number of images in each split, and percentage of genuine cannabis and non‐cannabis images. We report results on each split and consider the average on the testing sets as the final accuracy. All models were trained from scratch, since available pre‐trained network backbones were trained on large image datasets that consist almost exclusively of natural scenes, unlike the microscopic images used in this study. The software package was implemented in the Python programming language, version 3.8, importing libraries from Pytorch 1.8.0 and CUDA 10.1, and executed on a NVIDIA GTX 1080Ti graphic card for GPU acceleration.

**TABLE 1:** Data stats. Number of images in the train, validation, and test subset partitions in the two data splits used for the software experiments.

	Split 1				Split 2			
	Train	Validation	Test	Total	Train	Validation	Test	Total
Genuine cannabis	1020	131	135	1286 45.5%	1008	133	145	1286 45.5%
Non‐cannabis plant material	1241	148	151	1540 54.5%	1237	151	152	1540 54.5%
Total	2261 80.0%	279 9.9%	286 10.1%	2826 100.0%	2245 79.5%	284 10.0%	297 10.5%	2826 100.0%

### 4.1 CNN classification models

Both classification models, the basic CNN and the DLA, were trained for 80 epochs. We used a custom binary cross entropy loss function with equal weights for each class, and the standard Adam optimizer [51] with a 10^−4^ learning rate. The batch size selected was the largest possible for the GPU's memory, 16 for the CNN model and 6 for the DLA model. Training augmentations included geometric transformations – rotations, translations, scaling and horizontal flip, and texture transformations – brightness, hue, saturation, gamma, and contrast. All hyper‐parameters values are specified in the configuration files available in the authors' github repository. The model weights selected were those that yielded the highest prediction accuracy on the validation set. Validation was performed upon the end of each training epoch. Results were evaluated by the percentage of correct prediction of image labels on the test sets. Performance was measured by the average precision of the models' predictions on the two distinct data partitions. Table 2 presents results. Similar accuracies were achieved in the two distinct data splits. As expected, the DLA performed better than the basic CNN model, achieving 95.89% and 92.77% accuracies, respectively.

**TABLE 2:** Comparison of model performance. Results are evaluated by average prediction of correct labels on the test sets. The composite method, integrating object detection with image classification, yielded the highest accuracy.

Model	Type	Split 1	Split 2	Average
Basic CNN	Whole‐image classifier	91.26%	94.28%	92.77%
DLA	Whole‐image classifier	96.50%	95.29%	95.89%
YOLO	Object detector	93.00%	91.25%	92.12%
DETR	Object detector	83.00%	87.50%	85.30%
Composite method	YOLO + bounding box classifier	**98.25%**	**96.97%**	**97.61%**

*Note*: Best results achieved in each category are highlighted in bold.

### 4.2 YOLO and DETR object detection models

**FIGURE 8:**
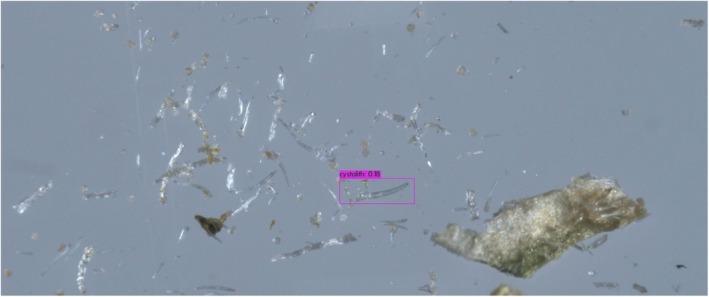
Correct YOLO detection of a cystolith from genuine cannabis material. YOLO outputs bounding boxes surrounding detected objects, each with a class label and a confidence value.

**FIGURE 9:**
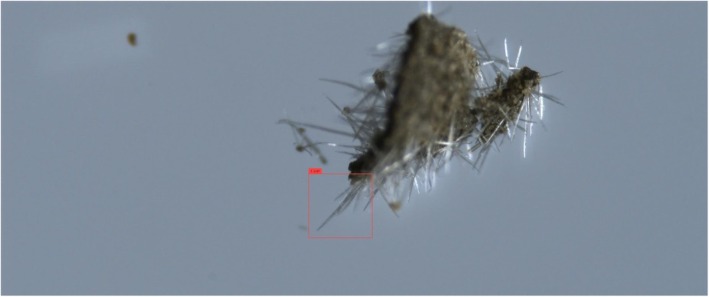
Correct DETR detection of non‐glandular trichome hairs on a non‐cannabis plant. DETR outputs bounding boxes surrounding detected objects, each with a class label and a confidence value.

In both object detectors, we used the default configurations provided by the software implementers, including network hyper‐parameters, loss functions, and optimizers. The only exceptions were the number of training epochs and the confidence threshold. Training deep neural networks is an iterative process, executed until performance on the validation subset does not significantly improve. The YOLO training process spanned over 6000 epochs, and the model's weights were selected based on the maximal mean average precision. The DETR training process spanned over 500 epochs, and the model's weights were selected based on minimal loss value. The confidence threshold is a value that determines the model's certainty in assigning a label to a detected object. In general, higher thresholds result in fewer detected objects, but with a higher probability that their assigned labels are correct, whereas lower thresholds result in more detected objects having lower confidence levels. After experimenting with a range of confidence values, we found that YOLO provided optimal detections (i.e., bounding box coordinate locations and correct labels) with a low‐confidence threshold, whereas DETR required a high threshold for high performance. For optimal results on the *validation* subsets, we used confidence threshold values of 0.05 and 0.3 for YOLO and DETR, respectively. An example of a good YOLO detection of a genuine cystolithic hair with a confidence of 0.18 can be seen in Figure 8. Figure 9 displays an example of a good DETR detection of a non‐cannabis plant hair with a confidence of 0.97. These specified confidence levels are on examples of detected trichomes taken from the *test* subset, provided for each detected object along with its class label.

Performance of the object detectors is measured as the percentage of correct predictions after the decision scheme described in Section 4.3 and depicted in Figure 6. “No detection” predictions are considered erroneous. For fair comparison, the exact same training/validation/test data partitions were used. Moreover, this decision scheme, although relying on the object detectors' output, predicts a binary classification label for the whole image, as do the classification models. Results, on the test sets, are presented in Table 2. Overall, YOLO achieved higher accuracy (92.12%) than DETR (85.30%). This was expected, as ViT‐based models are generally known to surpass CNN‐based models only when using networks that were pre‐trained on large datasets.

### 4.3 The composite method

**FIGURE 10:**
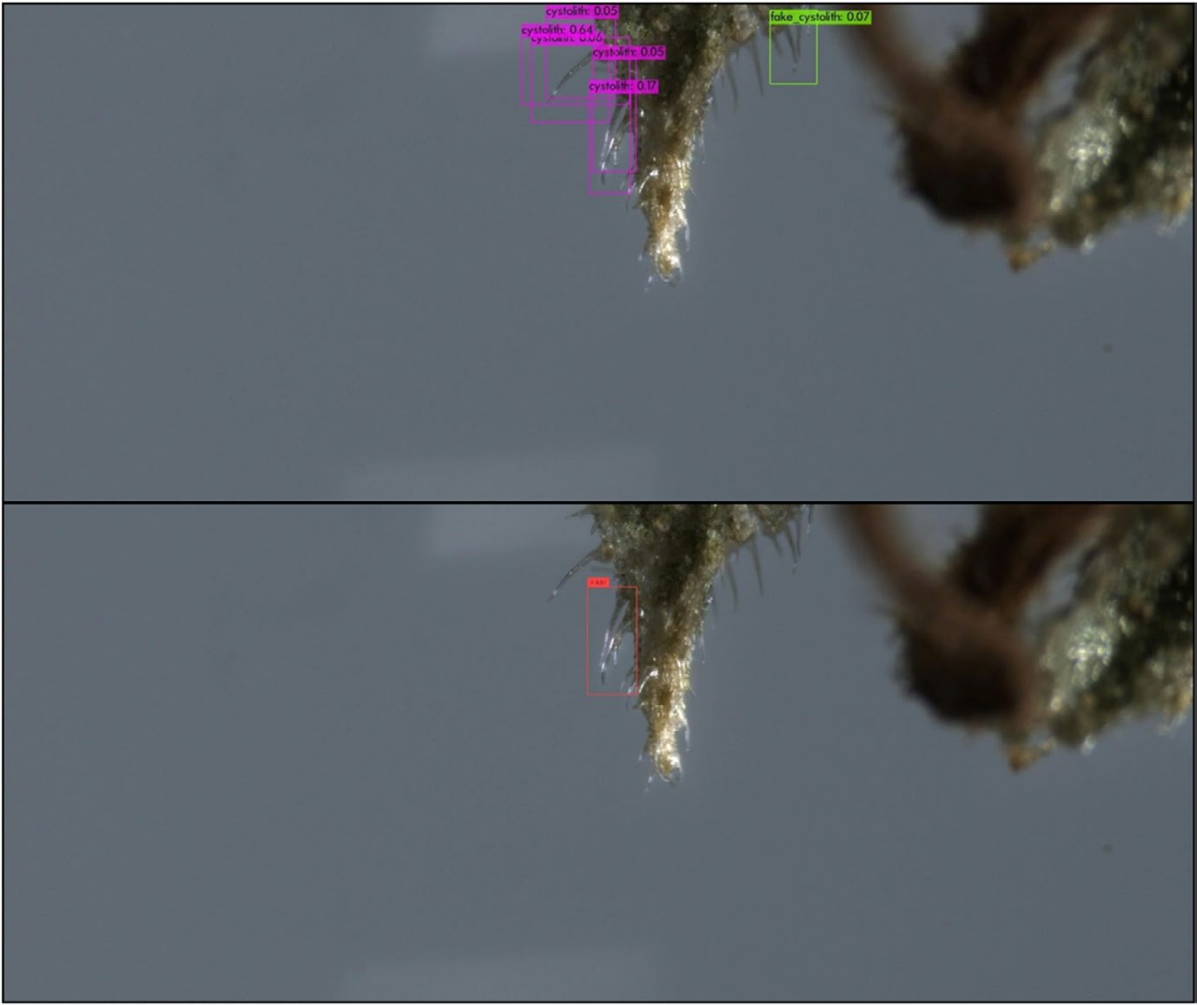
Example of outputs of the object detection models on a genuine cannabis image. Displayed are correct YOLO detections (top, magenta‐colored bounding boxes) that DETR detected erroneously (bottom, red‐colored bounding box). This examples showcases the use of a lower YOLO threshold, resulting in multiple true detections of the same cystolith, but also an incorrect detection (top, green‐colored bounding box).

The two versions of the composite method were tested with both YOLO and DETR as base detectors. Each base detector had the same configuration, hyper‐parameters, and trained weights, as those used when applying the object detector as a standalone method. The second‐stage image classifier is the same DLA network that was previously used as standalone classifier for whole images, with minor modifications. Since this classifier operates on detected objects from the first stage rather than whole images, the input size is smaller, and was further rescaled to 256 × 256 pixel patches. To optimize memory efficiency, the batch size was increased from 6 to 12, and the learning rate was reduced to 10^−5^. The smaller sized input affects the resolution, but not the structure, of the feature maps in the various layers, resulting in a reduced number of input nodes to the first fully connected layer, from 512 × 32 × 32 to 32 × 3 × 32 nodes. In the three‐stage strategy (Figure 7, right), the bounding boxes provided to the classifier are generated by the YOLO/DETR models, which were trained with lower confidence thresholds. The reasoning here is the preference for “no detections”, at this stage, thus allowing an opportunity for using the whole‐image classifiers, rather than erroneous detections that will eliminate the need for their usage, thus outputting incorrect labels. Results for all four composite methods are presented in Table 3. The training/validation/test data partitions used here are the same as in all previous experiments. The evaluation criteria are also the same, that is, the percentage of true classifications on the test subset. The composite methods that used YOLO had better accuracy compared with the methods using DETR. These results were to be expected, as YOLO also had better performance as a standalone object detector. An example of a good YOLO detection that DETR failed to detect correctly is shown in Figure 10. While YOLO detected mostly glandular hairs from the correct class (cannabis hairs), DETR wrongly detected a single hair as a non‐cannabis hair. The best performing method was the one that used YOLO with the bounding box classifier, with an overall accuracy of 97.61%. This method was chosen as the optimal composite method, demonstrating a significantly better performance compared with other approaches (Table 2).

**TABLE 3:** Performance comparison of different versions of the composite method. Results are evaluated by average prediction of correct labels on the test sets. Highest accuracy was achieved using YOLO as a first‐stage object detector on the whole image, followed by YOLO operating on bounding boxes obtained by YOLO with a lower threshold.

Method	Strategy	Split 1	Split 2	Average
DETR + whole‐image classifier	2‐stage	91.96%	96.63%	94.30%
DETR + bounding box classifier	3‐stage	90.56%	96.30%	93.43%
YOLO + whole‐image classifier	2‐stage	97.90%	96.63%	97.27%
YOLO + bounding box classifier	3‐stage	**98.25%**	**96.97%**	**97.61%**

*Note*: Best results achieved in each category are highlighted in bold.

## 5 CONCLUSION

This research was conducted with the aim of developing an automatic classification tool for identifying non‐glandular trichome hairs in cannabis and non‐cannabis plants. This tool is intended for use by police forensic departments and drug laboratories. First, we evaluated the ability of binary classifiers operating on microscopic images. Next, by selecting the discriminating criteria of trichome hairs, we trained modern object recognition models to detect them. Finally, using a combination of specially trained object detectors and whole‐image classifiers, we designed a novel method that was able to classify microscopic images with an accuracy of 97.61%.

The proposed method offers several practical benefits in forensic workflows. By integrating this system into routine evidence analysis, plant materials can be rapidly and reliably classified under a microscope equipped with an integrated camera. This approach can significantly reduce the reliance on labor‐intensive chemical tests, which are costly and time‐consuming. The system not only identifies whether trichomes in an image belong to cannabis or synthetic plant material but also highlights candidate trichomes and can provide confidence scores for each classification. This feature enhances its utility as a robust, reproducible, and efficient tool in forensic investigations.

We conclude that the application of deep learning and computer vision concepts can be beneficial in the domain of drug detection. With an efficient, low‐cost, and well‐performing method like the one proposed, the standard process of drug analysis can be significantly elevated. With more available training data, the model's accuracy could most likely be improved. We believe that this proposed method can be applied as part of routine forensic practice of identifying a plant as cannabis, which is considered a dangerous drug according to the Drug Ordinance in many countries.

## CONFLICT OF INTEREST STATEMENT

The authors declare no conflict of interest.
